# Overexpression of *MsSAG113* gene promotes leaf senescence in alfalfa *via* participating in the hormone regulatory network

**DOI:** 10.3389/fpls.2022.1085497

**Published:** 2022-12-08

**Authors:** Shuwen Li, Hong Xie, Lingfang Zhou, Di Dong, Yaling Liu, Chenyan Jia, Liebao Han, Yuehui Chao, Yinglong Chen

**Affiliations:** ^1^ School of Grassland Science, Beijing Forestry University, Beijing, China; ^2^ Inner Mongolia M-Grass Ecology And Environment (Group) Co., Ltd, Hohhot, China; ^3^ The University of Western Australia (UWA) Institute of Agriculture, and University of Western Australia School of Agriculture and Environment, The University of Western Australia, Perth, WA, Australia

**Keywords:** alfalfa, *MsC3H-39*, *MsSAG113*, RNA-seq, senescence

## Abstract

**Introduction:**

Alfalfa (Medicago sativa) is a kind of high quality leguminous forage species, which was widely cultivated in the world. Leaf senescence is an essential process in plant development and life cycle. Here, we reported the isolation and functional analysis of an alfalfa SENESCENCE-ASSOCIATED GENE113 (MsSAG113), which belongs to the PP2C family and mainly plays a role in promoting plant senescence.

**Methods:**

In the study, Agrobacterium-mediated, gene expression analysis, next generation sequencing, DNA pull-down, yeast single hybridization and transient expression were used to identify the function of MsSAG113 gene.

**Results:**

The MsSAG113 gene was isolated from alfalfa, and the transgenic plants were obtained by Agrobacterium-mediated method. Compared with the wildtype, transgenic plants showed premature senescence in leaves, especially when cultivated under dark conditions. Meanwhile, application of exogenous hormones ABA, SA, MeJA, obviously acclerated leaf senescence of transgenic plants. Furthermore, the detached leaves from transgenic plants turned yellow earlier with lower chlorophyll content. Transcriptome analysis identified a total of 1,392 differentially expressed genes (DEGs), involving 13 transcription factor families. Of which, 234 genes were related to phytohormone synthesis, metabolism and transduction. Pull-down assay and yeast one-hybrid assay confirmed that alfalfa zinc finger CCCH domain-containing protein 39 (MsC3H-39) could directly bind the upstream of MsSAG113 gene. In conclusion, the MsSAG113 gene plays a crucial role in promoting leaf senescence in alfalfa via participating in the hormone regulatory network.

**Discussion:**

This provides an essential basis for further analysis on the regulatory network involving senescence-associated genes in alfalfa.

## Introduction

Leaf senescence is the late stage in plant development, during which leaves change from pale yellow to yellow. During senescence, nutrients from old leaves are diverted to other parts of the plant to initiate new tissue, such as new leaves or developing organs and seeds (Jing et al., 2002). As a result, plant leaf cells undergo a series of changes in morphology, structure, and metabolism leading to leaf abscission, a process known as senescence syndrome (Bleecker and Patterson, 1997). Although senescence is an age-dependent process, it is often triggered by a range of biological and abiotic stresses (Gan and Amasino, 1997). External factors, such as environmental stress and long-term darkness may also induce leaf senescence, which is considered as a self-protection mechanism to cope with the stress to prevent crop yield or quality reduction (Chao et al., 2018).

Recently, the study of plant leaf senescence has been favored by a large number of researchers, and the related molecular mechanisms have also made good progress. In senescent leaves, the senescence-associated genes (*SAG*) are expressed, which are considered as a marker of cellular senescence (Wistrom and Villeponteau, 1992). Senescence associated genes have been cloned and identified in many plants. *OsSAG12-2* codes for a functional protease that negatively regulates stress-induced cell death in rice (Singh et al., 2016). A gene encoding an acyl hydrolase is involved in leaf senescence in *Arabidopsis thaliana*, chemically induced overexpression of *SAG101* causing premature senescence in both attached and isolated leaves of transgenic *Arabidopsis* plants (He and Gan, 2002). The senescence associated gene *AhSAG* under aluminum stress promoted senescence in transgenic plants and further induced or promoted the occurrence of programmed cell death (PCD) (Zhan et al., 2013). Growing isolated leaves in the dark can induce leaf yellowing and reduce chlorophyll content. Therefore, isolated leaves are used as models to study leaf senescence (Liebsch and Keech, 2016).

In addition, plant hormones play an important role in plant growth, development and metabolism. The senescence process is also regulated by various plant hormones such as salicylic acid (SA), abscisic acid (ABA), ethylene, jasmonic acid (JA), cytokinins, and auxin (He et al., 2002). Studies have shown that the ABA receptor gene *PLY9* has a role in aging old leaves and inhibiting the growth of young tissues (Zhao et al., 2016). There are many reports on the complex mechanisms of gene interactions regulating leaf senescence. *RhHB1* is a gene strongly over-expressed in senescent petals, which can be induced expression by ABA or ethylene. It was shown that ABA or ethylene treatment could induce increased *RhHB1* expression, while gibberellin GA_3_ treatment delayed this process (Lü et al., 2014). Leaf senescence is usually accompanied by a decrease in cytokinin content, but the increase of cytokinin content can alleviate leaf senescence (Balibrea Lara et al., 2004). In 2015, Surya et al. found that the expression of *converted structural genes* (*IPT*) encoding the cytokinin biosynthetic enzyme isoprenyl transferase delayed leaf senescence of the transgenic rapeseed plants under controlled environments (Kant et al., 2015).

Protein phosphatases of the PP2C type are monomeric enzymes present in prokaryotes and eukaryotes. PP2C is the largest family of protein phosphatases in plant species. Plant PP2C family proteins play important roles in transcriptional regulatory pathways such as stress response, ABA synthesis, and developmental signaling (Schweighofer et al., 2004). The *AHG1* gene of the PP2C protein family has special functions during seed development and germination (Nishimura et al., 2007). Two phosphatases 2C genes, *ABI1* and *ABI2*, negatively feedback the ABA signaling pathway (Merlot et al., 2001). *SAG113* belongs to the PP2C family and was founded to localize to the cis-Golgi as a negative regulator of ABA signaling, specifically inhibiting stomatal closure, further leading to senescence and eventual desiccation (Zhang et al., 2012).

Alfalfa (*Medicago sativa*)is an important leguminous herbage speceis, which is rich in protein, trace elements and vitamins. It has been widely used in the world because of its important ecological and economic values (Hrbáčková et al., 2020). Therefore, it is important to study the senescence of alfalfa leaves to improve its application value.

Although senescence associated genes have been widely reported in many different plants, only a few studies involved in alfalfa. Therefore, in this study the role of a senescence associated gene, *MsSAG113* was explored in the senescence regulatory network, aiming to lay a foundation for improving alfalfa quality by genetic engineering methods.

## Materials and methods

### Plant materials and growth conditions

Seeds of alfalfa (*Medicago sativa*) (cv. Zhongmu No. 1) were vernalized at 4°C for 4 d and cultivated under 16 h light (25°C) and 8 h dark (23°C) until root development. The seedlings were transferred to pots filled with mixed peat, vermiculite and perlite, and grown under 16 h light (25°C) and 8 h dark (23°C). It is difficult to control the developmental senescence, thus many senescence-related studies have been performed on isolated leaves in dark cultures or attached leaves in dark cultures alone (Pruzinská et al., 2005). For phenotype analysis of detached leaves, alfalfa leaves of 3-month-old wild type (WT) and *MsSAG113* transgenic plants were collected and immersed in 2-(N-Morpholino) ethanesulfonic acid hydrate (MES) buffer (3mM, pH 5.8) in the dark or with different plant hormone treatments.

### Subcellular localization

The SAG113-GFP-F/R primers (**Supplementary Table S1**) were used for the construction of subcellular localization vector *35S*::*MsSAG113*:*GFP* and the *35S*::*GFP* vector was used as a control. The recombinant plasmid was transferred into EHA105 *Agrobacterium tummefaciens* and injected into tobacco plants and cultured for subcellular localization. The position of green fluorescent protein (GFP) signal in the cells was observed with a confocal microscope (Leica TCS SP8, Leica Microsystems).

### Exogenous hormone treatments and gene expression

Two-month-old alfalfa plants were selected and sprayed with hormones involving 50 μmol/L ABA, 0.5 mmol/L SA, 10 μmol/L MeJA, 10 μmol/L 6BAP, respectively. The leaves were collected at 0 min, 10 min, 20 min, 30 min, 1 h, 3 h, 6 h, 9 h, and 12 h for expression analysis. Meanwhile, leaves at different developmental stage (1 month-old, 2 month-old and 3 month-old) were harvested for *MsC3H-39* gene expression analysis. Total RNA from the leaf samples of different treatments was extracted by a Plant RNA Kit (Omega Bio-tek, Inc., USA), and cDNA was synthesize using PrimeScript™ RT reagent Kit (TaKaRa, Japan) for real-time quantitative RT-PCR (qRT-PCR) with primer pair of SAG113-RT-F/R and C3H-39-RT-F/R (**Supplementary Table S1**). Expression of *MsSAG113* and *MsC3H-39* was calculated by the 2^-ΔΔCT^ method with three biological replicates (Schmittgen and Livak, 2008).

### Transformation of plants

The sequence of *MsSAG113* (GenBank Access No.: KT592510) was obtained from NCBI (National Center for Biotechnology Information) and analyzed in MEGA version 6.0. Two primers for SAG113-F/R (**Supplementary Table S1**) were used to clone the coding domain sequence (CDS) from alfalfa. Primer 3302Y-SAG113-F/R (**Supplementary Table S1**) was used to obtain plant expression vector *35S*::*SAG113*. The expression vector was then transformed into alfalfa mediated by *Agrobacterium transformation* (Hoekema et al., 1983). Three different lines (S1, S6 and S7) with high expression levels were selected for further experiments.

### Measurement of endogenous hormones and chlorophyll contents

Two-month-old transgenic and WT leaves with similar state were selected and endogenous hormones were detected by enzyme-linked immunoassay (ELISA) as described previously (Engvall and Perlmann, 1971). Chloroform was used to extract the chlorophyll content as previously reported (Matile et al., 1988). The absorbance of the extract was measured by spectrophotometer, and the content of each pigment in the extract was calculated. All samples were repeated three times.

### Next generation sequencing and analysis

Mature leaves of three independent lines were selected for transcriptiome sequencing and DEGs were identified by DESeq R package (version 1.18.0) (Robinson et al., 2009) with parameters: adjusted p-value <0.05 and |log_2_FC|≥1. Gene Ontology (GO) analysis of DEGs between samples was performed using GOseq R package (version: Release 2.12) (Young et al., 2010). KOBAS software (version: 2.0) was used for functional analysis of DEGs in the KEGG pathway (Mao et al., 2005).

### DNA pull-down

The potential *cis* elements of upstream of *MsSAG113* were investigated *via* the PlantCARE website (https://bioinformatics.psb.ugent.be/webtools/plantcare/html/). Based on the predicted result, one pair of primers SAG113-bition-F/R labeled with biotin (**Supplementary Table S1**) was synthesized and used for DNA pull-down. The alfalfa nuclear proteins were extracted by a nuclear protein extraction kit (Solarbio, China) and the extraction was incubated with the target sequence of *MsSAG113* containing biotin. Compound was pulled down with streptavidin magnetic beads (Beyotime, China) according to the manual. The compound products were washed five times, separated by SDS-PAGE, and identified by mass spectrometry.

### Yeast one-hybrid

Two pairs of primers, pAbAi-SAG113pro-F/R and pGADT7-C3H-39-F/R (**Supplementary Table S1**) were used for construction of vectors for yeast one-hybrid. Recombinant plasmids pAbAi-MsSAG113_pro_ and pGADT7-MsC3H-39 were co-transformed into yeast Y1H strain. Different concentrations of aureobasidin (AbA) (TaKaRa, Japan) were used to screen a suitable concentration for yeast one-hybrid, and transformed yeast cells were grown on SD/-Leu/AbA medium for further investigation.

### Transient expression of *MsC3H-39*

The *MsC3H* CDS was cloned with MsC3H-39-F/R primers (**Supplementary Table S1**) and ligased into 3302Y vector, resulting in plant expression vector. The recombinant plasmids were transiently transferred into 2 month-old alfalfa leaves with empty vector 3302Y as controls and the leaf phenotype was observed. After 48h of dark treatment, total RNA was isolated from each leaf sample, and the expression levels of *MsSAG113* was analyzed with qRT-PCR as described above.

## Results

### Location and expression pattern of *MsSAG113*

**Figure 1:**
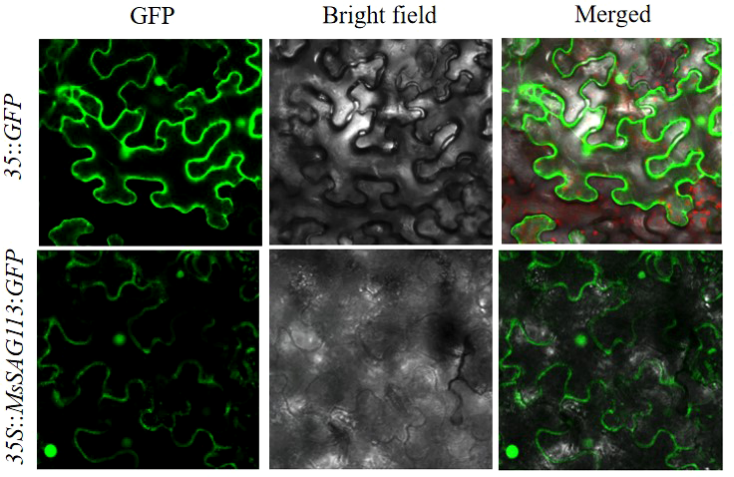
Subcellular localization of *MsSAG113* in the nucleus and cytoplasm detected by GFP signal. Scale bars =25 μm.

Based on the Cell-PLoc package and UniProt analysis, *MsSAG113* was predicted to be a nuclear-localized protein. The expression of *MsSAG113*-GFP fusion protein in tobacco leaves was captured showing strong GFP signal in the nucleus and cytoplasm (**Figure 1**), which indicated subcellular localization of *MsSAG113* protein was in nucleus and cytoplasm.

**Figure 2:**
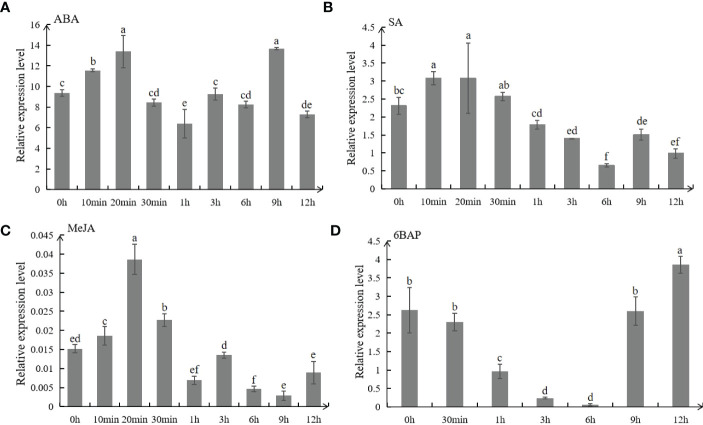
Expression analysis of *MsSAG113* in alfalfa by qRT-PCR. *MsSAG113* under different hormones treatments: 50 μM ABA **(A)**, 0.5 mM SA **(B)**, 10 μM MeJA **(C)**, and 10 μM 6BAP **(D)**. The values are means ± SD (n=3). In graphs, bars with different lowercase letters indicate significant difference (p<0.05).

All hormones used in this study had significant effects on the expression of *MsSAG113*,which was increased rapidly at first and then decreased with the passage of time in ABA,SA and MeJA treated plants. The expression of *MsSAG113* reached its peak at 20 min after spraying with ABA and MeJA hormones (**Figures 2A–C**). It indicated that the expression of *MsSAG113* gene was regulated by ABA, MeJA or SA in the short term. The expression of *MsSAG113* in plants treated with 6BAP showed a down-regulated trend within 6 h and then recovered rapidly at 9 h (**Figure 2D**). These results indicated that *MsSAG113* was involved in short-term regulatory of plant hormone network.

### The role of *MsSAG113* in premature leaf senescence

**Figure 3:**
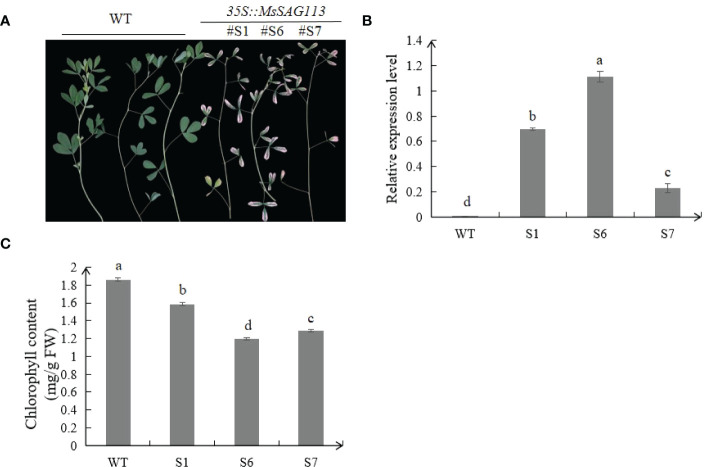
Phenotypes analysis of transgenic and WT alfalfa. Phenotype of three transgenic lines and WT alfalfa of 2 month-old **(A)**. Relative expression levels of *MsSAG113* in transgenic and WT plants **(B)**. Chlorophyll contents of three transgenic and WT plants **(C)**. The values are means ± SD (n = 3). In graphs, bars with different lowercase letters indicate significant difference at p<0.05.

Through the analysis of transgenic plants S1, S6 and S7, we found that under normal condition, transgenic plants exhibited early senescence phenotype and leaves turned yellow much earlier than those of WT plants. All three transgenic samples showed significantly higher expression levels of *MsSAG113* than those in WT plants (**Figure 3B**). Meanwhile, the chlorophyll contents in transgenic plants were drastically lower than those in WT (**Figure 3C**). These results indicated that *MsSAG113* promoted early senescence and chlorophyll degradation in alfalfa.

### Dark accelerates senescence of detached leaves of transgenic alfalfa

**Figure 4:**
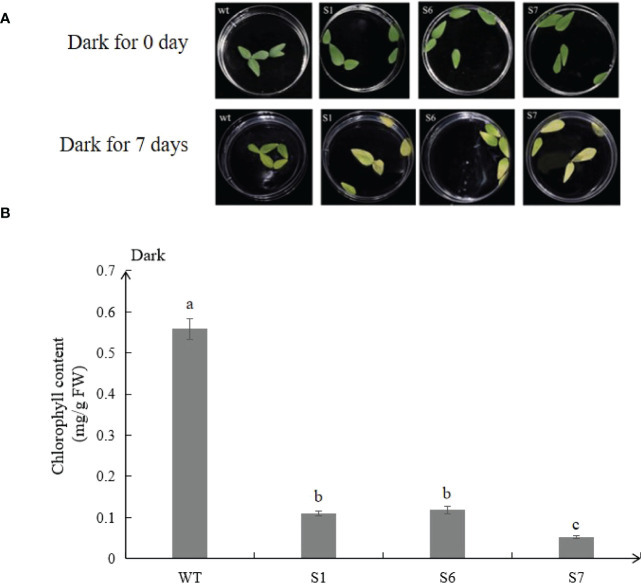
*MsSAG113* is involved in dark-induced leaf senescence. Phenotypic analysis of 2 month-old wild type and transgenic isolated leaves after dark treatment **(A)**. Chlorophyll contents of leaves under dark treatment **(B)**. The values are means ± SD (n = 3). In graphs, bars with different lowercase letters indicate significant difference at p<0.05.

The detached leaves from transgenic plants turned yellow much earlier than WT under continuous dark for 7 days (**Figure 4A**), and chlorophyll contents were consistent with the senescence phenotype. The chlorophyll content of transgenic lines S1, S6 and S7 was 19.6%, 21.1% and 9.3% of that of wild-type lines, respectively (**Figure 4B**).

### Detection of endogenous hormones in overexpressed plants

**Figure 5:**
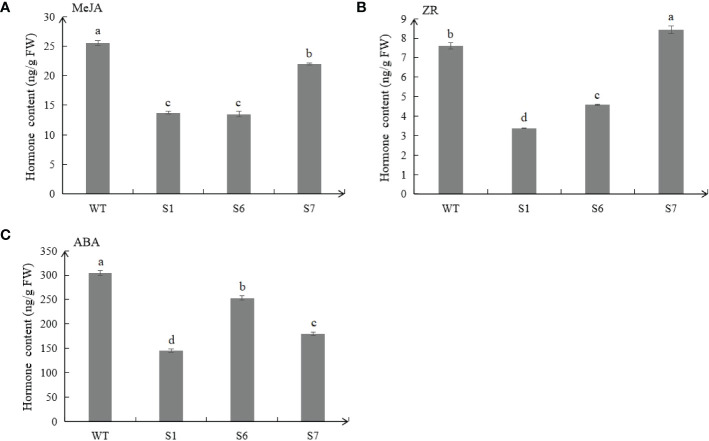
Endogenous hormone contents in transgenic and WT plants. Different hormones, including MeJA **(A)**, ZR **(B)** and ABA **(C)** contents in transgenic and WT plants.The values are means ± SD (n = 3). In graphs, bars with different lowercase letters indicate significant difference at p<0.05.

The contents of plant hormones in WT and transgenic plants were detected. Results showed that MeJA and ZR contents of transgenic plants were lower than those of WT (**Figures 5A, B**). In addition, ABA contents of the three *MsSAG113* transgenic lines were all lower than those in WT. Compared with WT, ABA content in S1, S6 and S7 plants was decreased by 53.3%, 16.7% and 41.2%, respectively (**Figure 5C**).

### Analysis of detached leaves of transgenic *MsSAG113* treated with exogenous hormones

**Figure 6:**
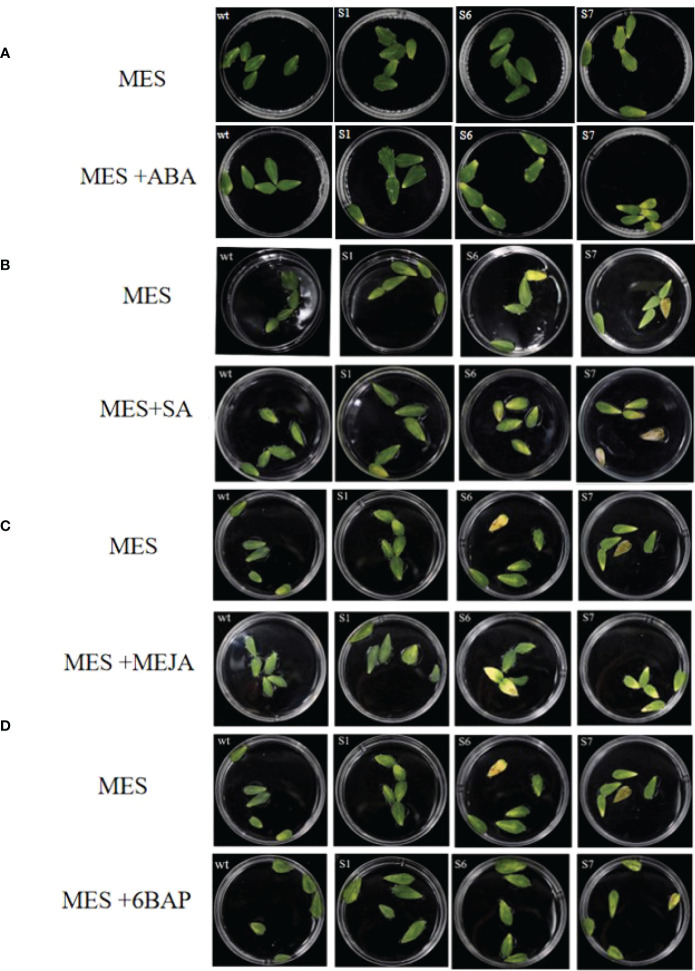
Detached leaves in MES with different hormones. Transgenic and WT detached leaves with or without ABA treatments for 5 days **(A)**. Transgenic and WT detached leaves with or without SA treatments for 8 days **(B)**. Transgenic and WT detached leaves with or without MeJA treatments for 12 days **(C)**. Transgenic and WT detached leaves with or without 6BAP treatments for 12 days **(D)**.

Detached leaves of S1, S6, and S7 treated with ABA, SA or MeJA showed obvious senescence on day 5, 8 or 12, respectively (**Figure 6**). Meanwhile, leaf senescence in S1, S6, and S7 leaves were also more pronounced than that in WT (**Figures 6A–C**). After 12 days of treatment with 10 μM 6BAP, the leaves of S1, S6 and S7 were significantly greener than those without *MsSAG113* gene in plant senescence, indicating that *MsSAG113* gene is involved in hormone network.

**Figure 7:**
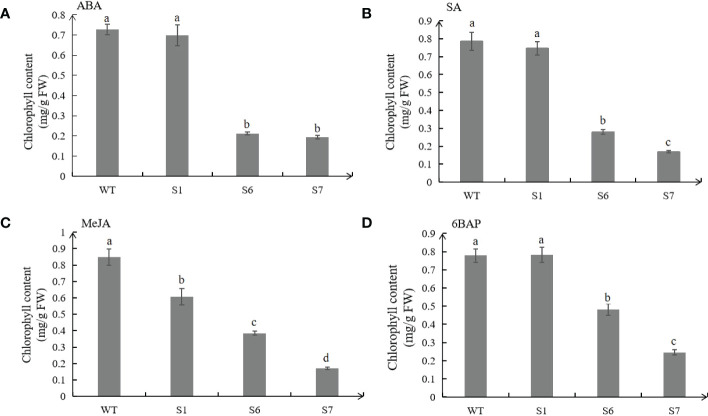
Chlorophyll contents in leaves of wild type (WT) and transgenic plants under hormone treatments. Chlorophyll contents of WT and transgenic leaves treated with 50 μM ABA for 5 days **(A)**. Chlorophyll contents of WT and transgenic leaves treated with 0.5 mM SA for 8 days **(B)**. Chlorophyll contents of WT and transgenic leaves treated with 10 μM MeJA for 10 days **(C)**. **C**hlorophyll contents of WT and transgenic leaves treated with 10 μmol/L 6BAP for 12 days **(D)**. The values are means ± SD (n = 3). In graphs, bars with different lowercase letters indicate significant difference at p<0.05.

Under the treatments of ABA, SA and MeJA, the chlorophyll contents of transgenic plants, especially in S6 and S7 were lower than those from WT, which were consistent with the senescence phenotypes (**Figures 7A–C**). Under 6BAP treatments, the chlorophyll contents of WT leaves were higher than those in S6 and S7 (**Figure 7D**). However, samples of transgenic S1 showed no significant difference in chorophyll contents with WT under different hormone treatments except MeJA.

### DEGs and TFs were identified by transcriptome analysis

**Figure 8:**
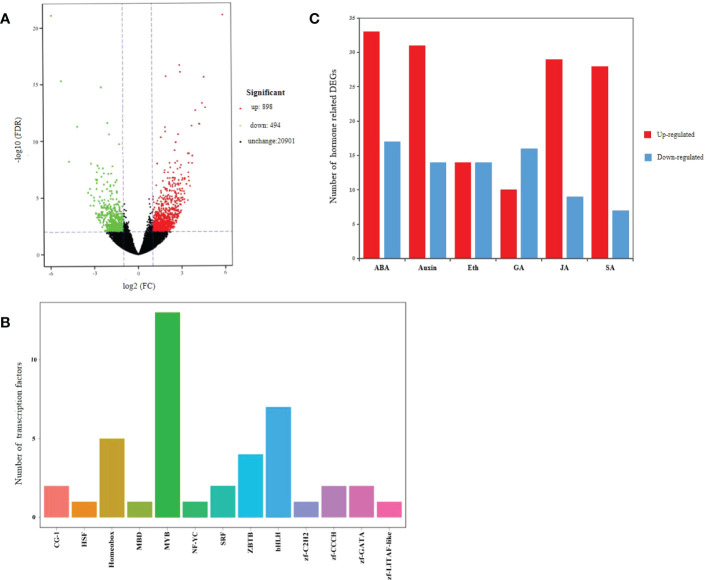
Transcriptome analysis of transgenic and WT alfalfa. Volcano plot of DEGs **(A)**. In graphs, each plot represents one gene with three colors, including red (up-regulated), green (down-regulated) and blank (unchanged). The X-axis represents the value of log_2_ (Fold Change) in the two samples, and the Y-axis indicates the negative value of log_10_ (FDR). Differentially expressed transcription factor genes identified with *MsSAG113* overexpression **(B)**. In graphs, the X-axis represents transcription factor family, and the Y-axis represents the number of transcription factor genes. Hormone related DEGs **(C)**. Eth indicates Ethylene. Red indicates up-regulated, and blue indicates down-regulated.

Transcriptome analysis identified a total of 1,392 DEGs in the transgenic and WT plants, of which 898 were up-regulated and 494 were down-regulated (**Figure 8A**; **Supplementary Table S2**). There were 42 differentially expressed TFs from 13 families identified, of which MYB (13), bHLH (7), Homeobox (5), ZBTB (4) and zf-CCCH (2) families were among the top 5 (**Figure 8B**; **Supplementary Table S3**). Meanwhile, 234 DEGs related to plant hormones were identified mainly involving ABA, Auxin, JA and SA, among which 145 were up-regulated and 77 were down-regulated (**Figure 8C**; **Supplementary Table S4**). These results showed that over-expression of *MsSAG113* gene affected multiple senescence associated genes in alfalfa.

### TF MsC3H-39 enhanced MsSAG113 expression

**Figure 9:**
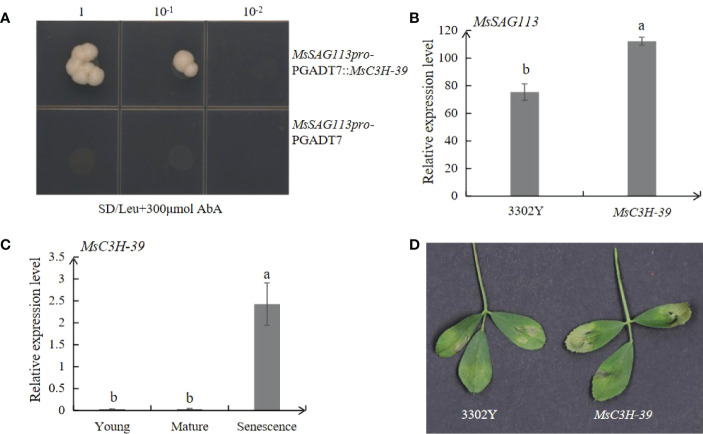
Interaction and expression analysis of *MsSAG113* with TF MsC3H-39. **(A)** Interaction analysis of upstream of *MsSAG113* and MsC3H-39 transcription factors. The number indicates dilution times of transformed yeast cells. **(B)** Relative expression levels of *MsSAG113* gene in alfalfa with transcient expression of empty vector or *MsC3H-39*. **(C)** Expression of *MsC3H-39* in young, mature and senescent leaves of alfalfa. **(D)** 3302Y empty vector and *MsC3H-39* transiently express the senescent phenotype in alfalfa leaves. The values are means ± SD (n = 3). In graphs, bars with different lowercase letters indicate significant difference at p<0.05.

By DNA pull-down, 54 target proteins were collected, including alfalfa zinc finger protein MsC3H-39, which was also found in transcriptome analysis. To verify whether the TF can recognize and change *MsSAG113* expression, we performed yeast one-hybrid and qRT-PCR analysis of alfalfa with transient expression of *MsC3H-39* gene. Yeast one-hybrid assay showed that only those yeast clones containing *MsSAG113* promoter and *MsC3H-39* gene could survive on selection medium (SD/-leu/AbA_300_) (**Figure 9A**). These results suggest that MsC3H-39 transcription factor can bind directly to the promoter of *MsSAG113*.

The expression of *MsC3H-39* in alfalfa leaves was the highest in senescence leaves indicating the involvement of *MsC3H-39* in plant senescence. Meanwhile, transiently expression of *MsC3H-39* result in apparent senescence symptoms compared with empty vector 3302Y (**Figures 9C, D**). Transient expression of *MsC3H-39* gene and qRT-PCR analysis of *MsSAG113* showed that *MsC3H-39* significantly increased the expression of *MsSAG113*, suggesting that MsC3H-39 transcription factor positively regulated the expression of *MsSAG113* by binding the upstream of the gene (**Figures 9B–D**).

## Discussion and conclusion

The regulatory network of senescence associated genes is constantly updated, and many *SAGs* were found being involved in gene expression regulation, signal transduction, macromolecular degradation and other senescence processes (He et al., 2001; Gepstein et al., 2003; Buchanan-Wollaston et al., 2005; Guo and Gan, 2012). *SAG113* belongs to the PP2C superfamily and plays an indispensable role in plant senescence. Senescence can limit crop yield and normal plant growth and development (Gan and Amasino, 1997). In this study, we explored the senescence regulation mechanism of *SAG113* gene in alfalfa, which could provide a reference for studying the growth and development mechanism of other legumes and improving crop yield.

Expression analysis showed that *MsSAG113* gene was affected by ABA, SA, MeJA and 6BAP hormone treatments. These hormones are considered to be the main internal factors that control the aging process of leaves (Balibrea Lara et al., 2004; Liebsch and Keech, 2016). It was reported that application of ABA-treated daffodils promoted senescence, and the increase in leaf ABA content was visually consistent with leaf senescence (Hunter et al., 2004). In *Arabidopsis thaliana* mutants deficient in SA signaling, the expression patterns of many genes were altered, and the mutants delayed yellowing and reduced necrosis (Morris et al., 2000). Exogenous application of JA led to premature senescence in leaves of wild-type *Arabidopsis thaliana*, but such effect absented in JA mutant plants (He et al., 2002). In our study, exogenous ABA, SA or MeJA accerlated early senescence of detached leaves with *MsSAG113* overexpression (**Figure 7**). These results indicated that ABA, SA and MeJA might participate in plant senescence regulation of *MsSAG113* gene. Abscisic acid promoted aging, while 6BAP generally inhibited aging (Smart, 1994). Early physiological studies have shown that exogenous cytokinin treatment of most monocotyledonous and dicotyledonous plant species could delay leaf senescence (Richmond et al., 1957). Our study achieved similar results. Exogenous 6BAP hormone treatment of isolated leaves indicated that leaf senescence was delayed, and the delay in transgenic leaf senescence was more obvious (**Figure 7**). Therefore, *MsSAG113* gene, as a senescence regulator, may have a role in regulating leaf senescence by participating in a hormonal regulatory network.

PP2C enzymes play an important role in signal transduction. Six groups of PP2C family protein phosphatases are considered to be negative regulators of ABA (Schweighofer et al., 2004). Previous studies showed that SAG113 is a negative regulator of ABA signaling pathway, which can inhibit stomatal closing in leaves and specifically involved in the control of water loss during leaf senescence (Wang et al., 2011; Zhang and Gan, 2012). Overexpression of *FsPP2C1* in *Arabidopsis* reduced the degree of seed dormancy and was insensitive to ABA, so *FsPP2C1* had a negative regulatory effect on ABA (Gonzalez-Garcia et al., 2003). This precisely corroborates our results that ABA contents were reduced in plants overexpressing *MsSAG113.* Therefore, it is reasonable to speculate that the increased transcription level of *SAG113* may be involved in negatively regulating ABA signaling, leading to the inhibition of ABA synthesis.

Compared with the wild type, the leaves of *MsSAG113* transgenic alfalfa had obvious yellowing, and the chlorophyll content was significantly reduced (**Figure 3**). These results are consistent with previous research. During senescence, chlorophyll was degraded, which was visually manifested as yellowing of leaves (Curty and Engel, 1996). Overexpression of *MYBR1* could delay leaf senescence in *Arabidopsis*. Loss-of-function *MYBR1* plants exhibited faster chlorophyll loss and senescence (Jaradat et al., 2013). Darkness was often thought to be a cause of senescence, in which the synthesis of chlorophyll was blocked during the reductive phase (Weaver and Amasino, 2001; Eckhardt et al., 2004). Our results showed that after 7 days of dark treatment, the color of the isolated leaves in wild type was greener than that of the transgenic plants, with about 10 times difference in chlorophyll content (**Figures 4**, **5**). These results suggest that *MsSAG113* gene be involved in the metabolic process of chlorophyll to regulate leaf senescence in alfalfa.

Our study showed that application of exogenous ABA, SA and MeJA significantly increased chlorophyll content in WT compared to transgenic plants. Previous studies on other plants observed the similar results (He et al., 2002; Lü et al., 2014; Zhao et al., 2016). However, we found that in isolated leaves treated with exogenous 6BAP, the chlorophyll content in S1 was increased compared with WT, while S6 and S7 were significantly reduced. Studies have shown that at the onset of senescence, under the activation of the *SAG12* promoter, feedback regulation of cytokinins occurs, which can be inhibited in delaying leaf senescence (Kant et al., 2015). The *SAG12* and *SAG13* promoters retarded the degradation of chlorophyll in isolated tomato senescent leaves under dark induction, while only *PSAG13::IPT* plants showed delayed chlorophyll degradation in isolated leaves and florets of broccoli (Chen et al., 2001; Swartzberg et al., 2006). Therefore, we inferred that under exogenous 6BAP treatment, the *SAG113* promoter was activated in S6 and S7 plants, resulting in feedback regulation of cytokinins, affecting chlorophyll synthesis, and thus reducing chlorophyll content of leaves. In addition, there was no significant difference in chlorophyll content between S1 plants and WT plants treated with exogenous hormones ABA, SA and 6BAP, which may be due to the different expression levels of *MsSAG113* in different transgenic lines and a phenotype in the transgenic plant is affected by gene expression levels and environmental factors.

RNA-Seq has become one of the important techniques for analyzing plant leaf senescence. Transcription factors and plant hormones are central to the senescence regulatory networks. In Arabidopsis, NAC, WRKY, AP2-EREBP and bHLH family transcription factors are regulated (Balazadeh et al., 2008). In this study, a total of 1,164 aging-related genes and 42 TFs from 13 families were identified, mainly including MYB, bHLH, Homeobox, ZBTB and zf-CCCH. The studies of plant senescence in *Arabidopsis thaliana* showed that many hormones were involved in the regulation of senescence in different ways (van der Graaff et al., 2006). By transcriptome analysis, we identified a total of 234 genes involved in hormone biosynthesis, metabolism, signal transduction and response. These results provided further evidence that *MsSAG113* gene plays important roles in regulating senescence in alfalfa and participating in hormone related regulatory network.

The identification of upstream regulators revealed the mechanism of *MsSAG113* gene in regulating senescence. This study screened the upstream regulatory protein of MsC3H-39 by DNA pull-down and verified it by yeast one-hybrid and transient expression analysis. MsC3H-39 protein belongs to CCCH-like of zinc finger proteins, which can be classified into C2H2, C2C2, C2HC, C2C2C2C2, C2HCC2C2 and CCCH types according to the number and order of zinc ion-bound Cys and His residues in the secondary structure of fingers (Sanchez-Garcia and Rabbitts, 1994; Klug and Schwabe, 1995; Mackay and Crossley, 1998). CCCH-like zinc lipoproteins have been shown to be involved in senescence regulation in previous reports. As a CCCH-type protein, OsDOS can delay leaf senescence, partially through the JA pathway (Jan et al., 2013). Two CCCH zinc finger proteins, AtC3H49 and AtC3H20 can delay leaf senescence and participate in ABA and JA responses in *Arabidopsis* (Lee et al., 2012). KHZ1 and KHZ2, two proteins of CCCH, positively regulated leaf senescence in *Arabidopsis thaliana* (Yan et al., 2017). The present study identified *MsSAG113* as a target gene of *MsC3H-39* by DNA pull-down assay and confirmed by yeast one-hybrid and qRT-PCR analysis. Meanwhile, we found that expression levels of *MsC3H-39* were the highest in senescence leaves, much higher than those in young and mature samples. Furthermore, MsC3H-39 can cause an earlier senescence phenotype and the senescence associated gene, *MsSAG113* can be induced by MsC3H-39 (**Figures 9A–D**). These data suggest that MsC3H-39 directly regulates the expression of *MsSAG113* and promotes leaf senescence.

**Figure 10:**
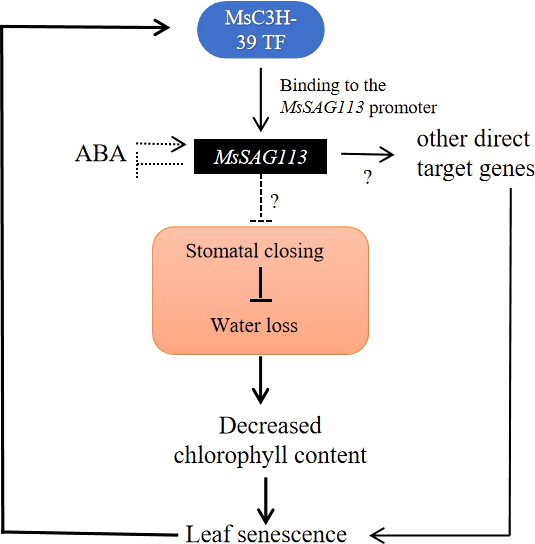
Potiential regulatory of *MsSAG113* in senescence in alfalfa. MsC3H-39 TF can enhance the expression of *MsSAG113* gene by recognizing and binding its promoter. ABA induces *MsSAG113* expression and *MsSAG113* negatively regulates ABA accumulation. *MsSAG113* may negatively regulate stomatal closure, resulting in accelerated water loss, a decrease in chlorophyll content and triggering plant senescence in alfalfa.

In summary, *MsSAG113* gene, as a senescence regulator, can be induced by hormones and regulates plant senescence in alfalfa by participating in the hormone regulatory network. MsC3H-39 directly recognizes and binds the upstream of *MsSAG113* and enhances its expression (**Figure 10**). Meanwhile, A total of 42 TFs associated with plant senescence and 234 genes involved in hormone regulation was identified by transcriptome analysis, which further indicated the important role of *MsSAG113* gene in leaf senescence and hormone regulation network in alfalfa. This study lays a foundation for studying the mechanism of alfalfa senescence and provides new insights for improving the growth and production in pasture legumes.

## Data availability statement

The data presented in the study are deposited in the NCBI repository. The accession numbers are PRJNA822614 and OP880214.

## Author contributions

YHC, LH, and SL conceived and designed the experiment. SL and LZ conducted the experiments. DD and HX analyzed the data. CJ and YL contributed analysis tools. YHC provided financial support. SL wrote the manuscript. YHC and YLC edited and revised the manuscript. All authors made significant contributions to the manuscript and approved the final version for publication.

## Supplementary material

Supplementary Table 1 List of primers.

Supplementary Table 2 Differentially expressed genes.

Supplementary Table 3 Involved in hormone regulation genes in DEGs.

Supplementary Table 4 Transcriptional regulators in DEGs.
